# Spontaneous Ovarian Hyperstimulation Syndrome in a Triplet Pregnancy

**DOI:** 10.1155/2012/189705

**Published:** 2012-10-08

**Authors:** Nisha Rani Agrawal, Garima Gupta, Kusum Verma, Neeraj Varyani

**Affiliations:** ^1^Department of Obstetrics and Gynecology, Institute of Medical Sciences, Banaras Hindu University, Uttar Pradesh, Varanasi 221005, India; ^2^Department of General Medicine, Institute of Medical Sciences, Banaras Hindu University, Uttar Pradesh, Varanasi 221005, India

## Abstract

Ovarian hyperstimulation syndrome (OHSS) is a potentially life-threatening complication, usually iatrogenic after ovulation induction. OHSS is a very rare event in spontaneously conceived pregnancies. Only few cases have been reported in literature to the best of our knowledge. We report a very rare case of spontaneous critical OHSS (according to classification of severity of OHSS as mentioned in Greentop guidelines no. 5, 2006) associated with triplet pregnancy in a 26-year-old woman suffering from severe abdominal pain, distension, and dyspnea. Our case highlights the importance of a strong suspicion for OHSS when a clinical presentation could not be explained by common medical conditions.

## 1. Introduction

Ovarian hyperstimulation syndrome is a rare and potentially life-threatening complication of ovarian stimulation. OHSS is in almost all cases an iatrogenic complication, the incidence of which is increasing world-wide through an increase in controlled ovarian hyperstimulation cycles.

OHSS has a broad pathophysiologic spectrum ranging from mild illness to severe disease. As many as 33% of in vitro fertilization (IVF) cycles have been reported to be associated with mild forms of OHSS [1]. More severe OHSS has been reported in 3.1–8.0% of IVF cycles [1]. Women without any pharmacological intervention are rarely diagnosed with spontaneous OHSS [2] which needs prompt evaluation and management. Risk factors for OHSS include young age, low body weight, polycystic ovarian syndrome, higher doses of gonadotropins, and previous episodes of hyperstimulation. 

Even after an extensive search of literature, we were unable to find the incidence of critical spontaneous OHSS which makes our case even more significant. Symptoms of OHSS do not cease immediately after pregnancy, but may persist or even worsen during days after treatment [3] as in our case. Misdiagnosis as neoplasm may result in inadvertent interventions. Nevertheless, alternative diagnoses should always be considered, such as complication of an ovarian cyst (torsion, haemorrhage), pelvic infection, intraabdominal haemorrhage, malignancy, ectopic pregnancy, and appendicitis.

Thus knowledge and prompt recognition of ovarian hyperstimulation are essential for its prevention and management.

## 2. Case Presentation

A 26-year primigravida at 6 weeks of gestation presented with severe abdominal pain, distension, and dyspnea for the past 1 month, which was slow in onset and gradually progressing. The patient conceived spontaneously and denied having ever taken any ovulation inducing agent. Menarche commenced at age of 13 years and subsequently she had oligomenorrhea. She had a history of primary infertility of 8 years for which she was neither investigated nor was on any treatment. There was no history of fever, cough, chest pain, palpitation, jaundice, melena, hematemesis, decreased urine output, altered bowel habit, antitubercular treatment, joint pain, rashes, photosensitivity, oral ulcers, loss of appetite, vaginal bleeding, treatment for infertility, hyperemesis, headache, visual blurring, weight loss, or tremors. Her past and family history was not significant. On examination she was conscious, oriented, afebrile, pale and ill looking, weight: 78 kg, pulse: 110/min and regular, blood pressure: 126/80 mm Hg, and respiratory rate being 26/min. Icterus, clubbing, cyanosis, and pedal edema were absent. Lymph nodes were not palpable. Thyroid and breast examination were normal. Abdomen was distended with abdominal girth of 97 cm. Tense ascites and fluid thrill were present. There was no organomegaly. Pelvic examination revealed congested cervix. Uterus and ovaries could not be palpated because of tense ascites. Chest examination suggested signs of bilateral pleural effusion.

With this clinical presentation following conditions were considered as provisional diagnosis:

- Koch's abdomen,
- Malignancy,
- Collagen vascular disease.

**Figure 1:**
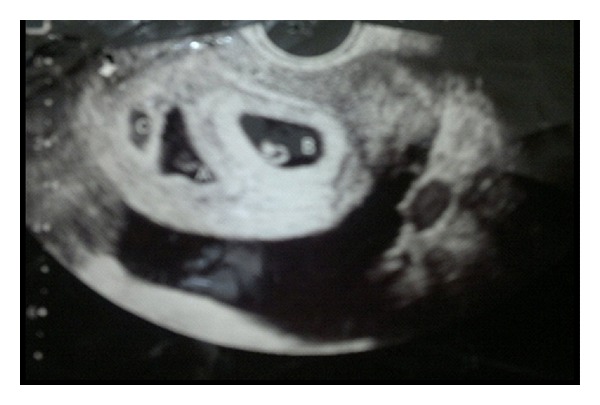
Ultra Sonography showing three gestational sacs with gross ascites.

On investigating, patient had anemia (Hb-88 gm/L, Hct-0.27), leucocytosis (14.5 × 10^9^/L) with polymorphs predominance. Her platelet count, chest X-ray, liver, renal, thyroid function tests, EKG, collagen profile, hormonal assays, and coagulation profile were normal. Ascitic tap examination revealed transudative picture. Aspiration cytology was negative for malignant cells and culture was negative for bacteria and fungi. Input output charting was 2.2 L/1.2 L. Abdominal sonography revealed 3 intrauterine gestation sacs of 6wk2d, 6wk3d, and 4wk3d, respectively. Right ovary was enlarged 8.2 × 7.4 × 8.3 cm with multiple large cysts and severe ascites with bilateral pleural effusion was present, suggestive of Ovarian HyperStimulation Syndrome (Figure 1). 

A diagnosis of critical OHSS was made. Her management was carefully tailored considering her pregnancy and severity of OHSS. Conservative management was initiated. Body weight, abdominal circumference, input/output, serum electrolytes, ultrasonography, and renal functions were closely monitored on daily basis. Later ultrasound-guided abdominal paracentesis was performed 3 times, in view of respiratory distress and increasing abdominal girth. Medical termination of pregnancy was done on patient's request at 10 weeks of gestation. Subsequently she developed pyoperitoneum which did not resolve with conservative management. Therefore she underwent exploratory laparotomy for drainage of pyoperitoneum and pus was sent for culture and sensitivity testing. Dense adhesions with septations were present. There was no evidence of uterine or intestinal perforation. Polycystic right ovary of approximately 8 × 6 cm adherent to parietal abdominal wall was present, which reconfirmed our diagnosis of critical OHSS*. E. coli* was grown in pus culture which was sensitive to ceftriaxone. Antibiotics were prescribed accordingly. Following this patient's general condition improved and is in our followup. 

## 3. Discussion

In this case study spontaneous OHSS in association with triplet pregnancy of 6-weeks of gestation was observed which is a very rare occurrence and requires documentation.

In 1996 Olatunbosun et al. [4] first reported case of severe spontaneous OHSS associated with pregnancy and polycystic ovarian disease to result in live births. In this case study patient had polycystic ovaries, thus sharing a common background of excess ovarian follicle growth as in our case study. 

The etiology and pathophysiologic characteristics of OHSS are poorly understood. OHSS is a systemic disease resulting from vasoactive products released from hyperstimulated ovaries. It is characterized by increased capillary permeability, leading to leakage of fluid from the vascular compartment, with third space fluid accumulation and intravascular dehydration [5]. Various factors including estrogen, histamine, prostaglandins, aldosterone, renin, and angiotensin II have been implicated in the development of this condition. Recent studies show high renin-like activity and elevated angiotensin II immunoreactivity in both plasma and ascitic fluid (angiotensin II being 6–9 fold higher than plasma) [6]. These findings are in favor of ovarian origin of the elevated renin-like activity and angiotensin II immunoreactivity in ascitic fluid of severe OHSS and suggest a stimulatory role of human chorionic gonadotropin (hCG) on the ovarian renin-angiotensin system during severe OHSS.

One third of the patients developing OHSS after IVF had no previous risk criteria. Exogenous and/or endogenous hCG is suggested as an etiologic factor. Recent studies have revealed the phenomenon of spontaneous OHSS in pregnancy to be caused by a mutation in FSH receptor which tends to recur in subsequent pregnancies [7].

Differential diagnosis includes disorders such as hyperreaction luteinalis, luteomas of pregnancy, and theca lutein cyst.

Management of OHSS is tailored according to its severity. Each case should be classified [8] as in Table 1.

Management guidelines for OHSS as per Green-top Guidelines no. 5 available from http://www.rcog.org.uk/womens-health/clinical-guidance/management-ovarian-hyperstimulation-syndrome-green-top-5 are as follows.

Hospital admission should be recommended to women with severe OHSS. Women should be kept under review until resolution of the condition. Multidisciplinary assistance should be sought for all women with critical or severe OHSS who have persistent haemoconcentration and dehydration. Features of critical OHSS should prompt consideration of the need for intensive care. Pain relief is best provided with paracetamol and if necessary oral or parenteral opiates. Nonsteroidal anti-inflammatory agents are not recommended. Antiemetic drugs used should be those appropriate for the possibility of early pregnancy, such as prochlorperazine, metoclopramide, and cyclizine. Women admitted to hospital with OHSS should be assessed at least daily, with more frequent assessment of those with critical OHSS. 

### 3.1. Inpatient Monitoring of Patients with OHSS as in Table 2

Allowing women to drink according to their thirst represents the most physiological approach to replacing volume. Women with severe OHSS with persistent oliguria and haemoconcentration despite initial colloid volume expansion may need invasive monitoring and should be discussed with an anaesthetist. Diuretics should be avoided as they deplete intravascular volume, although they may have a role with careful haemodynamic monitoring in cases where oliguria persists despite adequate intravascular volume expansion and a normal intra-abdominal pressure. Paracentesis should be considered in women who are distressed due to abdominal distension or in whom oliguria persists despite adequate volume replacement. Paracentesis should be performed under ultrasound guidance to avoid inadvertent puncture of vascular ovaries distended by large luteal cysts. Intravenous colloid replacement should be considered for women who have large volumes of ascitic fluid drained. Pelvic surgery should be restricted to cases with adnexal torsion or coincident problems requiring surgery and only undertaken by an experienced surgeon following careful assessment. Women should be reassured that pregnancy may continue normally despite OHSS, and there is no evidence of an increased risk of congenital abnormalities.

## 4. Conclusion

Our case highlights the importance of a strong suspicion for OHSS when a clinical presentation could not be explained by common medical conditions. Early identification of OHSS in a patient can improve the quality of life. If left untreated, OHSS can result in serious health complications and even death.

## Figures and Tables

**Table 1:** 

Grade	Symptoms
Mild OHSS	Abdominal bloating
	Mild abdominal pain
	Ovarian size usually <8 cm

Moderate OHSS	Moderate abdominal pain
	Nausea/vomiting
	Ultrasound evidence of ascites
	Ovarian size usually 8–12 cm

Severe OHSS	Clinical ascites
	Oliguria
	Haemoconcentration >0.45
	Hypoproteinemia
	Ovarian size usually >12 cm

Critical OHSS	Tense ascites
	Haemoconcentration >0.55
	White cell count >25 × 10^9^/L
	Oligo/anuria
	Thromboembolism
	Acute respiratory distress syndrome

**Table 2:** 

Assessment	Measurements
History and Examination	Pain
	Breathlessness
	Hydration
	Weight
	Heart rate, blood pressure
	Cardiovascular
	Abdominal girth, distension, ascites
	Intake and output chart

Investigations	Full blood count
	Haemoglobin, haematocrit, and white cell count
	Urea and electrolytes
	Liver function tests
	Baseline clotting studies
	Pelvic ultrasound (for ascites and ovarian size)
	Chest X-ray or ultrasonography (if respiratory symptoms)
	ECG and echocardiogram (if suspect pericardial effusion)
